# Microbial Biotechnology 2020; microbiology of fossil fuel resources

**DOI:** 10.1111/1751-7915.12396

**Published:** 2016-08-10

**Authors:** Ian M. Head, Neil D. Gray

**Affiliations:** ^1^ School of Civil Engineering and GeosciencesNewcastle UniversityNewcastle upon TyneNE1 7RUUK

## Abstract

This roadmap examines the future of microbiology research and technology in fossil fuel energy recovery. Globally, the human population will be reliant on fossil fuels for energy and chemical feedstocks for at least the medium term. Microbiology is already important in many areas relevant to both upstream and downstream activities in the oil industry. However, the discipline has struggled for recognition in a world dominated by geophysicists and engineers despite widely known but still poorly understood microbially mediated processes e.g. reservoir biodegradation, reservoir souring and control, microbial enhanced oil recovery. The role of microbiology is even less understood in developing industries such as shale gas recovery by fracking or carbon capture by geological storage. In the future, innovative biotechnologies may offer new routes to reduced emissions pathways especially when applied to the vast unconventional heavy oil resources formed, paradoxically, from microbial activities in the geological past. However, despite this potential, recent low oil prices may make industry funding hard to come by and recruitment of microbiologists by the oil and gas industry may not be a high priority. With regards to public funded research and the imperative for cheap secure energy for economic growth in a growing world population, there are signs of inherent conflicts between policies aimed at a low carbon future using renewable technologies and policies which encourage technologies which maximize recovery from our conventional and unconventional fossil fuel assets.

## Abstract 2

A perspective on the potential future roles of microbiology in the fossil fuel sector is presented. This is placed in the context of technical and economic factors that may facilitate or mitigate against the uptake of new technologies.

## Global energy futures and fossil fuels

In the long term, the global energy economy will be driven by renewable energy. However, progress to this sustainable future as outlined in the United Nations 2030 Agenda for sustainable development (UN, 2015) is relatively slow. The proportion of our energy that is derived from fossil fuels is only projected to fall from around 80% currently, to around 75% by 2030 (IEA, 2015a). Nevertheless, total energy demand is set to increase in the same period (IEA, 2015a). The International Energy Authority estimates that annual global energy demand will rise from 156 million GWh (2012 figures) to somewhere between 170 and more than 175 million GWh by 2020, and further to between 175 million and almost 200 million GWh by 2030 (IEA 2015b). The lower values are based on the assumptions required to maintain atmospheric CO_2_ levels below the 450 ppm level that would give a 50% probability that global temperatures would rise by 2°C or less (IEA 2015b). Thus, even with a reduced proportion of our energy coming from fossil fuels, oil consumption is set to increase, and peak at 99 million barrels per day by 2030 (IEA 2015a); an increase of around 9–10% from the present day. Natural gas consumption could increase as much as 30% in the same period (IEA 2015a), with beneficial effects on CO_2_ emissions, but with little effect on our overall reliance on fossil energy. It is therefore inevitable microbiological aspects of fossil fuel exploration and production and the environmental impacts that go hand‐in‐hand with our addiction to fossil energy, will continue to be of considerable practical importance and will need to be underpinned by both fundamental and applied research to 2020 and beyond.

Consideration of technological advances involving microbiology must, however, be placed in the wider context of the economics of fossil fuel recovery. Oil prices have been comparatively low throughout most of the 20th century, fluctuating between 20 to 40 dollars a barrel. This pattern gave way to an era from the early 1970s onwards of fluctuating but inherently higher prices especially in the most recent decade and a half. Rather than forcing the development of low carbon alternatives to fossil fuels, such recent high oil prices have driven the development technologies for recovery unconventional fossil fuel resources that are difficult and therefore expensive to extract. It remains to be seen how lower oil prices since 2014 (caused by an economic downturn, oversupply from conventional production and the success of now established and efficient unconventional recovery technologies) will persist and hence change the future energy and technology innovation landscape. It also remains to be seen how global and regional government policies will dictate the direction of future energy landscapes as they try to address the reality of population growth, the development necessary for the eradication of poverty and the need to limit carbon emissions.

This perspective will explore some of the grand challenges in the microbiology of fossil fuels and speculate about the role of microbiology and microbial biotechnology as we transition from fossil fuel reliance to a new energy future.

## Grand challenges

Ensuring secure global energy supply while minimizing environmental impact is one of the most pressing challenges facing the human race as we move towards 2020 and beyond. Research on a broad front is tackling that challenge and it will be met with contributions from the physical, chemical and biological sciences as well as from the engineering required to translate fundamental science into an operational reality. Research and engineering do not operate in a vacuum and social, economic and political considerations will all ultimately shape our future energy system (Larter and Head, 2014). Microbiology impacts many aspects of petroleum exploration and production and is therefore likely to influence and contribute to the transition from fossil fuels to renewable energy by offering reduced emissions pathways to fossil energy recovery and potentially technologies for carbon capture and utilization (CCU). As conventional oil and gas reserves dwindle and the rate of new discoveries declines we will increasingly rely on unconventional fossil energy sources such as heavy oil, shale oil, coal bed methane (CBM), shale gas and even methane hydrates (IEA, 2013).

Microbiology has much to offer in terms of potential new technologies that facilitate energy recovery from such systems while minimizing their carbon footprint. In addition to energy recovery, microbiology also affects operational aspects of petroleum production through mediating reservoir souring and corrosion and the potential impact of biofouling on flow assurance. These are long‐standing issues in the petroleum industry and there is scope for future microbiological innovations to improve their control. With respect to the environmental impact of fossil fuel exploitation and the technologies to mitigate this impact very little is yet understood about the influence of microbiology. For instance, we currently know little of the potential for favourable and unfavourable pH‐microbe‐mineral‐CO_2_ interactions in deep storage facilities such as saline aquifers, depleted oil and gas reservoirs and even porous basaltic rocks which are likely to each have resident microbial communities (Gniese *et al*., 2014).

### Transition from fossil fuels to renewable energy

As we transition to a low carbon energy economy it is essential that we develop new approaches for harnessing the energy from fossil fuels with reduced environmental impact. This need is predicated on the recommendations of the Intergovernmental Panel on Climate Change that total global CO_2_ emissions from fossil fuels between 2011 and 2050 need to be restricted to somewhere between 870–1240 Gt (Clarke *et al*., 2014). The simple expedient of increasing the amount of energy produced from natural gas can contribute to reduction in CO_2_. Generation of 1 kWh of electricity from natural gas produces just over 0.5 kg of CO_2_, which is approximately 70% of the amount of CO_2_ emitted from oil per kWh of electricity generated and 60% of the equivalent CO_2_ emissions for coal (IEA; https://www.eia.gov/tools/faqs/faq.cfm?id=74&t=11). Indeed between 1973 and 2014 the proportion of global electricity generation from gas has increased from around 12% to almost 22% representing an increase from around 170 TWh to over 5000 TWh and this trend is projected to continue in the future (IEA, 2015b). However, this alone will not be sufficient to keep CO_2_ emissions within the 870–1240 Gt target range (McGlade and Elkins, 2015). Thus, new lower emissions technologies to augment the implementation of renewable energy supplies will be necessary. This will include carbon capture and storage and CCU technologies as well as novel processes that tackle the problem of reducing emission as part of the fossil fuel recovery process. It has been suggested, for example, that the only realistic way to reach 2050 cumulative emissions targets is to keep fossil carbon in the ground (McGlade and Elkins, 2015). In essence, this means replacing the energy that would have been generated from burning that fossil carbon with energy from other energy sources. An alternative scenario that is tempting to consider is recovery of the energy trapped in the fossil carbon while retaining at least a proportion of carbon in the ground. As we discuss below, microbiological processes could potentially address this challenge.

### Conventional fossil fuels to unconventionals

Unconventional sources of fossil fuels are typically more expensive to produce than conventional reserves. This makes their exploitation highly sensitive to oil price fluctuations. Generally unconventional reserves are extracted using physical processes (e.g. hydraulic fracturing for CBM or thermal processing such as steam assisted gravity drainage – SAGD – for viscous heavy oil). Thermal processes in particular are costly due to the energy input required to recover the fossil fuels and for subsequent processing to facilitate transport and refining. This additional energy input clearly has potential implications for carbon emissions. There is therefore scope for less energy intensive microbial processes for recovering energy from these resources. Unconventional fossil fuels encompass heavy oil and oil sands, shale oil and gas, CBM and methane hydrates. There is a potential microbial dimension to the recovery of most of these assets, but heavy oil and CBM will be the focus here.

Heavy oil is crude oil with high viscosity (usually > 10 cP), and high specific gravity. The American Petroleum Institute (API) classifies heavy oil on the basis of API gravity (paradoxically the lower the API gravity the heavier the crude). Crude oils with an API gravity below 22.3° are considered as heavy oils. Other characteristics of heavy oils are low hydrogen‐to‐carbon ratios, high asphaltene, sulphur, nitrogen and heavy‐metal content, as well as higher total acid number. These characteristics make heavy oil difficult and costly to produce, transport and refine. However, heavy oil also dominates the global oil inventory. Hein *et al*. (2013), estimate that global bitumen and heavy oil resources are around 5.6 trillion barrels (bbl), the largest deposits found in the Orinoco heavy oil belt in Venezuela and the Western Canada Sedimentary Basin. As more readily and economically recoverable light oil reserves become depleted, heavy oil is increasingly being exploited for energy production. When considering the climate impact of heavy oil production (e.g. from the Canadian oil sands), it is estimated that greenhouse gas emissions are, on average around 2–3 times greater than for conventional oil production. However since the bulk (60–80%) of all greenhouse gas emissions from any oil, heavy and conventional alike, comes from combustion of the fuel in our vehicles and power stations the full life cycle emissions from heavy oil are only 10‐20% greater than from conventional oil (Charpentier *et al*., 2009; Bergerson and Keith, 2010).

The likelihood of increased reliance on unconventional hydrocarbon resources such as oil sands and heavy oil has spawned a range of technologies to improve production. Enhanced recovery using energy intensive approaches such as SAGD are being augmented with a range of cold recovery techniques involving displacement of oil with a range of gaseous or liquid injectants, so called miscible flood approaches (Shah *et al*., 2010). In addition chemical flood approaches, such as polymer and surfactant floods designed to alter interfacial tension between oil and reservoir sediments enhancing oil sweep and recovery have also being developed and are tested on a large scale (Shah *et al*., 2010). Moreover thermal techniques such as *in situ* combustion or fire flooding, which generate lower emissions and use less water than more conventional thermal processes such as SAGD have been used to enhance the recovery of heavy oil (Shah *et al*., 2010) and curiously it has even been proposed that nuclear power could provide low emissions energy for electrically heating oil sands for enhanced oil recovery (Donnelly and Pendergast, 1999), for example, an option that would reduce emissions compared to SAGD.

*In situ* catalytic upgrading, using a range of Ni‐, Co‐, Mo‐ and Pd‐based catalysts, linked to thermal process to alter oil properties in the reservoir, and facilitate production of higher quality oil from heavy oil reservoirs, is being explored experimentally (Shah *et al*., 2010). There is growing interest in the use of nanoparticulate catalysts in this field (Guo *et al*., 2015; Franco *et al*., 2016) and microbially deposited Pd nanoparticles (Omajali *et al*., 2015) have been shown to have some benefits over physically prepared nanoparticulate Pd with respect to their application for *in situ* catalytic upgrading and oil recovery (Hart *et al*., 2016).

The first reports of the capability of methanogenic consortia to convert alkanes to methane (Zengler *et al*., 1999) prompted the possibility that this process could be harnessed for enhanced recovery of energy from residual oil in reservoirs where conventional secondary and tertiary recovery was no longer feasible (Parkes, 1999). Subsequent demonstrations that methane production from crude oil as a substrate provided further credibility to this notion (Townsend *et al*., 2003; Gieg *et al*., 2008; Jones *et al*., 2008). There are several attractive features of this approach to fossil energy recovery. The fact that methane is ultimately the energy vector produced by the process means that it offers reduced CO_2_ emissions per unit of energy generated compared with oil and gas (see *Transition from fossil fuels to renewable energy*), moreover because methanogenic degradation of oil (or any organic substrate for that matter) produces both CO_2_ and methane, which have very different physical and chemical properties, there is potential to recover the methane while retaining the CO_2_ in the subsurface, for example, by maintaining slightly alkaline conditions, reducing the net emissions of fossil carbon for the process.

There have been a number of attempts to take this technology from the laboratory to practical application (Larter *et al*., 2005; Pfeiffer *et al*., 2008). Rates of microbial methane generation from light oil of up to 72 μmol/dm^3^/day have been reported (Wang *et al*., 2012), while rates of methanogensis from heavy oil and oil sands are 1 to 2 orders of magnitude lower. Consequently it seems that oil conversion into methane for enhanced energy recovery from heavy oil at least, may not be economically feasible. Moreover, the time at which such studies were initiated coincided with a global economic downturn and a slump in natural gas prices due to massive production of shale gas resources, one of the consequences of which was reluctance to invest in novel technology when the product, methane, had a low market value, providing an excellent example of how the development and implementation of novel technologies relies as much on prevailing economic and political conditions, as it does on sound science.

Similar principles have provided the basis for microbially enhanced production of CBM. A number of commercial ventures have been established to implement this technology on a large scale (e.g. Luca Technologies, Ciris Energy and Next Fuel, Transworld Technologies) with promising results (Ritter *et al*., 2015). As with enhancing methane generation from residual oil microbially enhanced coalbed methane production strategies have relied on stimulation of the resident microbiota by addition of inorganic nutrients (Ritter Vinson *et al*., 2015). However unlike crude oil conversion into methane where it is clear that methanogenic alkane degradation is a primary source of methane, the electron donors for microbially enhanced coalbed methane formation are unclear and it seems that despite extensive methane generation with coal substrates a relatively small amount of the coal mass is converted to methane. It seems likely that at least some microbial methane production from coal involves the generation of methanogenic substrates either from sorbed organic compounds such as fatty acids, alkanes and low molecular weight aromatics or from low molecular weight organic compounds generated from complex organic matter in coal (Orem *et al*., 2010). Methanol or other C‐1 compounds may also be an important substrate for coalbed methanogenesis (Dawson *et al*., 2012; Guo *et al*., 2012). Nevertheless how low molecular weight microbially degradable compounds are generated from coal remains unknown.

Recent work suggests an alternative mechanism of microbial methane formation from coal. Beckmann and her co‐workers have demonstrated that crystals of the phenazine dye neutral red can enhance methane generation from organic and inorganic substrates, including coal (Beckmann *et al*., 2016). This seems to occur not as a result of neutral red crystals acting like conductive wires, directly transferring electrons from coal to methanogens, but by a considerable increase in the reduction potential of crystalline neutral red compared with the soluble material. This enhances the harvesting of electrons from the coal, and soluble neutral red in equilibrium with the crystals shuttles electrons to the methanogen. It is proposed that the neutral red may shuttle electrons to a hydrogenase that generates hydrogen that can then be used to reduce CO_2_ to methane (Beckmann *et al*., 2016).

### Microbiology and fracking

No forward look focused on the microbiology of fossil fuel resources would be complete without consideration of shale gas. The drive to recover the huge amounts of natural gas trapped in very low permeability shales has had global impact on the economics of energy as well as raising considerable environmental concerns (Osborn *et al*., 2011; Jackson *et al*., 2014).

Pore sizes in shale are such (nm scale) that it is unlikely that the bulk of native shales host active microbial communities. Nevertheless, naturally occurring fractures in shale deposits do provide a more hospitable environment for microorganisms. Moreover, hydraulic fracturing to release trapped methane also most likely provides opportunities for microbial growth by increasing fluid movement and consequently delivering substrates that may support microbial growth and activity. There are presently few published studies on the microbial ecology of shale gas systems (Cluff *et al*., 2014; Jiménez and Krüger, 2014). From the sparse data currently available, it is apparent that the microbiota of shales is consistent with the chemistry of the shale‐associated aquifers (Cluff *et al*., 2014) with communities that have been characterized from the Marcellus Shale in Pennsylvania being dominated by a range of halophilic taxa.

While under *in situ* conditions, shales will harbour small populations of microorganisms, this does not mean that microbiology can be ignored when developing shale gas resources. Indeed it would be remiss to do so as the very processes that we use to enhance gas recovery are also likely to stimulate the native microbiota and provide conditions that are conducive to the activity of microorganisms introduced with fracking fluids themselves. Thus, as in the exploitation of any subsurface geological resource, we are likely to see problems with a microbiological dimension affecting shale gas production. For example, microbially influenced corrosion (MIC) and souring may arise in shale gas production facilities, depending on the prevailing conditions, and it may yet prove feasible to manipulate the properties of shale gas formations through management of microbial activity. For example, porosity and permeability of tight gas formations could be manipulated through degradation of organic matter in the shale, or microbially mediated mineral precipitation or dissolution.

### Microbially enhance oil recovery revisited

The concept of microbially enhance oil recovery (MEOR) which involves manipulation of microbial populations to change reservoir properties, for example, selective plugging, reduction in permeability, reduction in interfacial tension, has been with us for almost a century, though the general principles of MEOR were first formalized by ZoBell (1947). Despite this long history and the fact that many of the major oil companies, specialized service companies and academic researchers have explored MEOR at laboratory and field scale (Rassenfoss, 2011; Babcock *et al*., 2014; Kotlar *et al*., 2014; Dourado *et al*., 2015; Patel *et al*., 2015), MEOR does not seem to have been used extensively in practice. There is some skepticism in the oil industry about the efficacy of MEOR (Lazar *et al*., 2007; Patel *et al*., 2015).

Skepticism about the available MEOR technologies in part stems from the difficulties in attributing increases in oil production specifically to MEOR in field trials. First, it can be difficult to have robust control treatments when operating at field scale. Moreover, in practice it is difficult to ensure that other interventions are not implemented during an MEOR field trial as operators continually strive to improve daily production (Rassenfoss, 2011). Perhaps the biggest factor driving skepticism about the technology is the fact that there is a multitude of mechanisms that could explain enhanced oil recovery, and tests of MEOR tend to be somewhat empirical. There is almost nothing known about the effects of MEOR treatment on the microbiological, physical, chemical or mineralogical characteristics of a reservoir formation subject to MEOR in the field. Consequently when MEOR works, it works, but when it does not, there is no robust theoretical framework that allows the cause of failure to be clearly determined. This has led one commentator to state ‘MEOR has been looked at for decades; none of the reputable scientists back it. There have been no validated results other than lab tests in tubes’. (Rassenfoss, 2011). A proprietary MEOR system has been tested at field scale in Southwest Kansas, an Albertan oilfield and a Californian oilfield by a partnership of energy companies and a technology company developing MEOR technologies (Bauer *et al*., 2011; Havemann *et al*., 2015; Karicherla *et al*., 2016). The results have been promising, but not completely predictable and in the Kansas field the enhanced production was primarily limited to a single well. Nevertheless, despite issues of variability in production with time, evidence was provided for an increase in oil production for a sustained period, above the predicted production decline curve, leading to around a 2.5‐fold incremental increase in production (e.g. Havemann *et al*., 2015). While the technology is proprietary, its basis is reported to be inorganic‐nutrient stimulated oil biodegradation by organisms indigenous to the reservoir. An important part of the process is initial testing to demonstrate that the native microflora have the capacity to degrade a proportion of the oil under the conditions of the MEOR treatment (Havemann *et al*., 2015). The precise basis for this MEOR technology has not been revealed, but the inference from the published results from these field trials suggests that it relies on a much more precise understanding of the response of the indigenous microbial communities to amendment with the inorganic nutrients used in the process than has been the case in previously deployed MEOR strategies. This fundamental understanding of the microbial community response may be an important contributor to the promising results reported to date and may herald a new era of MEOR, which relies less on trial and error and more on basic understanding of the microbial catalysts responsible. With the staggering cost of exploration in frontier areas such as the Arctic and deepwater provinces, along with the sensitivity of these areas and presently low oil prices, it seems likely that there will be a greater focus on understanding the resident microbiota of mature petroleum reservoirs nearing the end of their conventional production lifetime in an effort to maximize the recovery of oil from already discovered resources.

### A place for bioelectrochemical systems?

The bulk of what has been discussed above relies on reworking and improving existing concepts. In the past these approaches may not have been ready for successful deployment due to a lack of tools to fully understand the complex microbiological and ecological processes that we have tried to manipulate while blindfolded with one hand tied behind our technological back. Economic and political conditions also may have stifled the development of some of the technologies at a point when they were otherwise practically deployable. With the world having changed in terms of the economic, environmental, social and political landscape of the oil industry, and the development of powerful new technologies to measure and understand complex microbial ecosystems like petroleum reservoirs, the limitations of past attempts to develop microbial technologies for enhanced energy recovery from fossil fuel reserves may now be overcome.

However, there are also frontier technologies that have been little‐explored in the context of the microbiology of fossil fuels. One of these emerging areas is the potential for application of bioelectrochemical technologies for energy recovery from fossil fuels. This approach, in principle, has the potential to recovery energy directly as electricity while leaving carbon in the ground.

With growing knowledge of new processes in microbial ecosystems such as direct intercellular electron transfer (Rotaru *et al*., 2014) and the demonstration that there is a relationship between the ability to enter into syntrophic relationships based on direct intercellular electron transfer, and the ability to respire solid phase electron acceptors, including electrodes (Rotaru *et al*., 2015) it is apparent that there may be considerable capacity in the microbial world to couple oxidation of organic matter to electrode respiration and hence electricity generation.

It has been speculated that this could form the basis for a large scale bioelectrochemical system for harvesting electricity directly from petroleum reservoirs (Head *et al*., 2014). In the conceptual model proposed, anodes would be inserted in to the organic carbon rich reservoir sediments, or most likely in the oil water transition zone where oil exists surrounded by a continuous water phase. Microbial oxidation of crude oil components would then be linked to anode‐reduction with the anode connected to some form of air cathode at the surface. This could potentially generate energy while leaving the oxidized carbon in the reservoir. However there are significant practical and technical barriers that would need to be overcome to achieve this, not least of which are the low current densities typically achieved in microbial fuel cells, and electrical losses due to resistance in the wiring of such a cell, not to mention the enormous electrode spacing which would lead to prohibitive internal resistance in the system (Head *et al*., 2014). Other factors that would need to be overcome to render such a system feasible would likely be formation specific. For example, oxidation of reduced iron on the anode could lead to the precipitation of iron oxides on the electrode, or in a sulphide‐rich system sulphide oxidation may lead to deposition of elemental sulphur for example, on the surface of the electrode leading to its passivation or fouling and thus reduced capacity for anode respiration (Daghio *et al*., 2016).

Alternative scenarios for conversion of the energy stored in fossil fuel reservoirs into electricity, while retaining carbon in the ground are also being envisaged. Rather than directly harvesting electrons from oil in the subsurface Steve Larter and Marc Strous at the University of Calgary are exploring the possibility of oxidizing oil *in situ*, coupled to reduction in a soluble electron shuttle which can be transported to a surface facility where the reduced electron shuttle is oxidized in a microbial or chemical fuel cell or alternative energy harvesting system, with the re‐oxidized energy shuttle being re‐injected into the oil‐bearing formation (Larter *et al*., 2016; http://www.ucalgary.ca/prg/research/project-1-syzygy). Oxidation of oil *in situ* offers the possibility of retaining the oxidized carbon underground resulting in a nominally zero emissions electricity generation system. Potential electron shuttling energy vectors include sulphate/sulphide, manganese and iron redox pairs or organic electron shuttles such as quinones. While a sulphate/sulphide‐based system has the attraction that the system is known to work at reservoir scale, through the phenomenon of reservoir souring, issues such as sulphide‐related corrosion, sulphide toxicity and the potential for anode passivation by deposition of intermediate oxidized sulphur species would need to be addressed. Nevertheless, there are a wide range of potential redox pairs that could be explored with a key consideration being generation of a reduced electron shuttle, that is, sufficiently stable and has a sufficiently negative reduction potential to allow for effective energy harvesting at a surface facility.

### Souring and corrosion control

Souring and corrosion are multimillion dollar problems in the petroleum industry. Most strategies for their control involve measures to control the activity of sulphate‐reducing microorganisms either by physical removal of sulphate from injection waters, nitrate treatment to mitigate against the activity of sulphate reducers by a number of potential mechanisms (Hubert, 2010) or application of broad‐spectrum biocides such as glutaraldehyde (Gieg *et al*., 2011). Recent research has highlighted the potential of some novel strategies for control of sulphide generation in petroleum reservoirs. Foremost among these is (per)chlorate treatment which has been shown to effectively control sulphide generation in pure cultures and laboratory model systems (Liebensteiner *et al*., 2013, 2014; Engelbrektson *et al*., 2014). Moreover, high throughput screening tools more commonly associated with drug‐discovery programmes are now being applied to identify potential new control agents for reservoir souring, holding promise for the identification of highly selective and potent new agents that may ultimately applied in the field (Carlson *et al*., 2015).

## A roadmap to 2020

Laying out a roadmap to the future in any field is a daunting prospect, if not foolhardy. History tells us that the most significant advances emerging from scientific and technical ingenuity are rarely predicted *a priori*. It would serve us well to heed the wise words of the Nobel laureate Nils Bohr ‘Prediction is very difficult, especially if it's about the future’. The following should therefore be treated with appropriate skepticism.

Globally, the human population will be reliant on fossil fuels for energy and chemical feedstocks for at least the medium term. While there has long been an appreciation that microbial activity is important in many areas relevant to both upstream and downstream activities in the oil industry, microbial technologies have struggled for recognition in a world dominated by geophysicists and engineers. In circumstances where oil prices are relatively low, the diminishing returns on large investments in exploration, especially in challenging frontiers such as the Arctic and deepwater provinces, mean that conventional prospecting approaches become less attractive. This restriction is likely to direct focus on maximizing the output from existing resources which may include microbial based approaches. Microbiology has often struggled to provide simple, reliable solutions that can be applied at an appropriate scale to problems faced in oil production. This is in part due to conservative attitudes towards untested technology and the fact that the major technologies that have sustained production in the oil industry offer primarily physics‐based solutions. However in the endeavour of extending production from mature basins that are reaching the end of their conventional production lifetimes, physical means are approaching their limits. Paradoxically, the very same drivers that may lead to a sharper focus on existing fossil fuel resources, also predicates against investment in innovative, yet potentially risky technology. Nevertheless, at the time of writing it was reported that US oil reserves surpassed those of Saudi Arabia and Russia, primarily due to technological advances that have provided access to previously unproducible resources.

In such a conservative and economically challenging landscape for the unconventional exploitation of fossil fuel resources, it is perhaps easier to predict what will NOT transpire, than what will. For example, it may be difficult to implement some of the more imaginative proposals for energy recovery from fossil fuel resources in the short term. Biolectrochemical energy recovery either directly or through the use of novel electron shuttles, is therefore unlikely to feature in energy futures with a 2020 horizon. Whether this will be true on longer timescales may be an altogether different prospect.

Any look to the future cannot ignore synthetic biology which is already generating important advances in diagnostics, the production of artificial enzymes, flavour and fragrance chemicals, dietary supplements (omega‐3 fatty acids) and in engineering plants for pollutant degradation. Large scale deployment of microorganisms generated using synthetic biology techniques in open environments, such as would be required for applications in enhanced energy recovery from fossil fuel resources are however substantially more challenging and it would not be unreasonable to conclude that synthetic biology solutions will not have a major role in the development of more sustainable routes to energy recovery from fossil fuels. Indeed, alternative low carbon energy sources may well be deployed at scale before synthetic biology has the opportunity to impact on energy production from fossil fuels.

Economics notwithstanding, recent scientific developments show some promise for larger scale application in energy recovery from coal and residual oil. Coal and oil conversion into methane, while not offering zero carbon emissions, does provide a fuel with a higher H:C ratio and hence higher energy density per mole, leading to reduced CO_2_ emissions from combustion‐based energy systems. Low‐cost gas from shale, may however lead to some delay in what are likely to be more expensive microbial solid‐ or liquid‐to‐gas conversion technologies.

There is a growing inventory of petroleum reservoir microbial community data facilitated by the wide and low‐cost availability of high throughput sequencing technologies. This is furnishing unprecedented information on microbial communities based on the analysis of both 16S rRNA genes and other marker genes as well as metagenomic analysis. While many contemporary studies of petroleum reservoir microbiota are to a degree descriptive, and on occasion illustrate some interesting correlations, to date there has been little systematic synthesis of such data to identify the factors that dictate the organisms that thrive under any particular set of reservoir conditions. More adept and sophisticated analyses of these data will first identify robust correlations and ultimately use these to develop testable hypotheses that will lead to a better understanding of how we can manage microbial populations in petroleum reservoirs to facilitate, for example, more reliable microbially enhanced oil recovery and/or control of souring. To maximize integration of available data, it is important that there is some standardization in petroleum reservoir microbiome studies and initial steps in this direction have been taken (Tsesmetzis *et al*., unpublished data). If we wish to test hypotheses about petroleum reservoir processes such as souring, robustly, it is essential that we do not rely solely on ‘omics techniques, powerful as they are, need to be integrated with well‐designed experiments to identify specific processes, measure their rates and undertake pure culture studies with relevant model organisms. The complex interplay between microbial activity and electrochemistry that characterizes MIC will make this a tougher nut to crack and advances in the control of MIC are likely to lag behind advances in souring control.

Application of novel agents for managing reservoir souring is likely to be tested at least at pilot scale and to a limited degree at field scale. This is likely to include testing of (per)chlorate, where there is already considerable momentum with the demonstration of potential advantages over nitrate‐mediated control of reservoir souring. New agents such as monofluorophosphate and other candidate compounds identified from high throughput screening are also likely to emerge as potential control agents to manage reservoir souring in a more selective manner.

A further major consideration with respect to the prospects for an increased role for microbiology in the fossil fuel industry is the source of development funding. With low oil prices and a focus of the oil industry on core business, industry funding in the area is likely to be hard won and recruitment of microbiologists is unlikely to be a high priority. Moreover there is a degree of schizophrenia in relation to publicly funded institutions with some government funding agencies actively promoting engagement with the oil and gas sector while others are more circumspect. In addition there is increasing pressure for universities and research organizations to distance themselves from fossil fuel‐related activities with the aim of promoting more sustainable alternatives. For example, at the time of writing the Fossil Free campaign's website (http://gofossilfree.org/commitments/) lists 48 universities that have divested from fossil fuel‐related investments. While it is important that developments in non‐fossil based energy should not be stifled, it is also clear that there is room for novel transition technologies that will reduce the environmental impact of our reliance on fossil energy which will, by necessity, persist in the next few decades.

## Conflict of interest

None declared.
